# Unraveling the neurotoxicity of titanium dioxide nanoparticles: focusing on molecular mechanisms

**DOI:** 10.3762/bjnano.7.57

**Published:** 2016-04-29

**Authors:** Bin Song, Yanli Zhang, Jia Liu, Xiaoli Feng, Ting Zhou, Longquan Shao

**Affiliations:** 1 Guizhou Provincial People’s Hospital, Guiyang 550002, China; 2 Nanfang Hospital, Southern Medical University, Guangzhou 510515, China

**Keywords:** autophagy, brain, DNA methylation, neurotoxicity, titanium dioxide nanoparticles

## Abstract

Titanium dioxide nanoparticles (TiO_2_ NPs) possess unique characteristics and are widely used in many fields. Numerous in vivo studies, exposing experimental animals to these NPs through systematic administration, have suggested that TiO_2_ NPs can accumulate in the brain and induce brain dysfunction. Nevertheless, the exact mechanisms underlying the neurotoxicity of TiO_2_ NPs remain unclear. However, we have concluded from previous studies that these mechanisms mainly consist of oxidative stress (OS), apoptosis, inflammatory response, genotoxicity, and direct impairment of cell components. Meanwhile, other factors such as disturbed distributions of trace elements, disrupted signaling pathways, dysregulated neurotransmitters and synaptic plasticity have also been shown to contribute to neurotoxicity of TiO_2_ NPs. Recently, studies on autophagy and DNA methylation have shed some light on possible mechanisms of nanotoxicity. Therefore, we offer a new perspective that autophagy and DNA methylation could contribute to neurotoxicity of TiO_2_ NPs. Undoubtedly, more studies are needed to test this idea in the future. In short, to fully understand the health threats posed by TiO_2_ NPs and to improve the bio-safety of TiO_2_ NPs-based products, the neurotoxicity of TiO_2_ NPs must be investigated comprehensively through studying every possible molecular mechanism.

## Introduction

Titanium dioxide nanoparticles, smaller than 1 μm in at least one dimension, possess specific physico-chemical characteristics [1] including antibacterial, ultraviolet-absorbing, photocatalytic, and self-cleaning properties [2]. Thus, TiO_2_ NPs are widely used in cosmetics, sun screens, ceramics, paints, packaging, lithium batteries, the food industry, and in medical applications [3]. However, the rapid development of nanotechnology and widespread applications of products containing TiO_2_ NPs have increased the risk of exposure. Therefore, numerous in vivo and in vitro studies have been performed to scrutinize the potential toxic properties of TiO_2_ NPs in recent years [4]. Research has demonstrated that TiO_2_ NPs can be detected in the main organs of experimental animals [5–6] and in exhaled breath condensate of exposed workers [7]. This accumulation can in turn damage affected organs and induce dysfunction.

The brain is of particular interest, as it is unable to regenerate from damage. Consequently, the neurotoxicity of nanomaterials should receive considerable attention. For this reason, we discussed the application and bio-distribution of TiO_2_ NPs, pathways through which they are translocated into the brain, harmful effects induced by them on the brain, and factors that can regulate their neurotoxic properties in our recent review [8]. However, the molecular mechanisms underlying this neurotoxicity were not discussed in detail. Therefore, in this review, we aim to discuss all previously described molecular mechanisms underlying the neurotoxicity of TiO_2_ NPs by summarizing published articles. From our research, we conclude that the major mechanisms are oxidative stress (OS), inflammatory responses, apoptosis, genotoxicity, and direct impairment of cell components. However, it appears that TiO_2_ NPs-induced neurotoxicity results from multiple mechanisms. Furthermore, other minor mechanisms exist and include disturbed distributions of trace elements, disrupted signaling pathways, dysregulated neurotransmitters and synaptic plasticity (Table 1 and Table 2).

**Table 1:** Mechanisms of neurotoxicity of titanium dioxide nanoparticles in in vivo studies.

objects	administration route	mechanisms of neurotoxicity	references

rats	intravenous injection	indirect mechanism (induced by cytokines and pro-inflammatory mediators in systemic circulation)	[6]
mice	nasal administration	inflammatory response (over-proliferation in glia cells)	[29]
rats	intravenous injection	OS and angiotensin system	[22]
rats	intravenous injection	multiple (OS, inflammatory response and DNA damage)	[55]
mice	oral administration	OS (ROS and anti-oxidant enzymes disturbed)	[23]
mice	inhalation	OS (H_2_O_2_ and MDA elevated)	[24]
mice	oral administration	other mechanisms	[57]
mice	intranasal administration	inflammatory response	[30]
pregnant rats	subcutaneous injection	OS	[26]
pregnant rats	oral administration	other mechanisms (cell proliferation inhibited)	[58]
pregnant mice	subcutaneous injection	other mechanisms (disrupted dopamine systems)	[59–60]
pregnant mice	subcutaneous injection	multiple mechanisms (apoptosis, OS and neurotransmitters)	[50]
mice	nasal instillation	OS	[21]
mice	nasal instillation	multiple mechanisms (OS and inflammatory response)	[49]
mice	delivery in abdominal cavity	OS	[25]
mice	intragastric administration	other mechanisms (disturbed distributions of trace elements, enzymes and neurotransmitters)	[61]
mice	intragastric administration	multiple mechanisms (apoptosis and OS)	[54]
mice	intranasal administration	P38-Nrf-2-mediated OS	[31]
neonatal rats	lactation exposure orally	disturbed synaptic plasticity	[62]
rats	trachea administration	inflammatory response	[32]
mice	injection in abdominal cavity	genotoxicity induced by OS	[43]

**Table 2:** Main mechanisms of neurotoxicity of titanium dioxide nanoparticles in in vitro studies.

cell types	mechanisms of neurotoxicity	references

BV2	OS (ROS, H_2_O_2_ elevated)	[19–20]
primary hippocampal neurons	multiple mechanisms (disrupted glutamate metabolism and dysregulated levels of NMDARs)	[56]
primary hippocampal neurons	apoptosis mediated by mitochondria- and endoplasmic reticulum-pathways	[35]
primary astrocytes	direct impairment of mitochondria and ROS	[38]
D384 and SH-SY5Y	direct impairment of mitochondria and cell membrane	[39]
SH-SY5Y	direct impairment of microtubules and cell morphology	[40]
SH-SY5Y	multiple mechanisms (changed cell cycle, apoptosis, and DNA damage)	[53]
C6 and U373	OS and impairment of mitochondria	[48]
C6 and U373	multiple mechanisms (inhibited cell proliferation, morphological change and apoptosis)	[52]
N9	apoptosis	[36]
U87	apoptosis	[37]
PC12	multiple mechanisms (OS and apoptosis)	[51]
PC12	other mechanisms (signaling pathway activated and arrested cell cycle)	[63]

Recent studies have reported that autophagy [9] and DNA methylation [10–11] are also involved in nanotoxicity (Table 3). Therefore, we hypothesized that autophagy and DNA methylation can be included as major mechanisms underlying the neurotoxicity of TiO_2_ NPs. Autophagy (from Greek, “auto” meaning oneself and “phagy” meaning to eat) was identified as a unique adaptation of cells to starvation and involves the cellular degradative pathway through which cytoplasmic cargo is delivered to the lysosome. Autophagy also acts as a dynamic recycling system wherein new building blocks and energy, for cellular renovation and homeostasis, are produced [12].

**Table 3:** Autophagy and DNA methylation in non-neuronal cells induced by TiO_2_ NPs.

cell type	mechanisms	references

normal lung cell	autophagy	[74]
primary human keratinocytes	autophagy	[75]
A549	DNA methylation	[100]

The literal meaning of “epigenetics” is “outside conventional genetics”, which is defined as all heritable alternations in gene expression. These stable alternations are not caused by changes to DNA sequence itself, but instead arise during development and cell proliferation [13–14]. DNA methylation is the one of the most extensively studied epigenetic mechanisms. Whether TiO_2_ NPs are able to induce neurotoxicity through altering autophagy or DNA methylation status in brain remains uncertain. Further studies are required to further verify their role in neurotoxicity induced by TiO_2_ NPs. To fully understand threats to the brain posed by TiO_2_ NPs, and improve the bio-safety of TiO_2_ NPs-based products, neurotoxicity of these compounds must be investigated comprehensively.

## Review

### Established mechanisms underlying the neurotoxicity of TiO_2_ NPs

To fully understand potential health threats posed by TiO_2_ NPs, we summarized recent articles about the neurotoxicity of TiO_2_ NPs in our recently published review. We found that after rats or mice were exposed to TiO_2_ NPs via several administration routes (e.g., nasal instillation, subcutaneous injection and oral exposure), NPs can be absorbed and translocated into the brain mainly through the blood–brain barrier (BBB) or the nose-to-brain pathway, which bypasses the BBB. Given that TiO_2_ NPs were able to pass the placental barrier and accumulate in the fetal brain by penetrating the undeveloped BBB, TiO_2_ NPs exposure during gestation can impair fetal brain development. Based on the limited excretion rate from brain, even low-dose exposure to TiO_2_ NPs can lead to gradual accumulation over a long period of time. This accumulation can in turn affect brain development, impair brain function, and can even result in disabilities in learning and memory assessed by poor performance in behavioral tests.

As stated previously, the major mechanisms of TiO_2_ NPs-induced neurotoxicity are oxidative stress (OS), inflammatory responses, apoptosis, genotoxicity, and direct impairment of cell components. However, in most situations, neurotoxicity occurs through multiple mechanisms. Furthermore, other minor mechanisms include disturbed distributions of trace elements, disrupted signaling pathways, dysregulated neurotransmitters, and synaptic plasticity.

#### Oxidative stress mechanism

Oxidative stress (OS) is defined as the production of reactive oxygen species (ROS) or/and reactive nitrogen species (RNS) at a rate much high than the elimination rate after the organism encounters harmful stimulus. OS can injure tissues and organs, and is often associated with diseases and aging. Meanwhile, oxidative stress, caused by NPs, is the most important and widely accepted mechanism of nano-neurotoxicity. ROS, such as superoxide, hydrogen peroxide, and hydroxyl radicals, are natural products of the regular oxygen metabolism [15–16]. However, these free radicals can interact within biological systems, resulting in oxidative damage to the organism. These harmful effects can be counteracted by biological antioxidants, including superoxide dismutase (SOD), catalase (CAT), glutathione peroxidase (GSH-Px), the expression of which needs to be coordinately regulated with the onset of OS [17–18]. If this balance is interrupted, levels of NPs-activated OS surpass the capacity of the biological antioxidants, potentially resulting in toxic oxidative stress. As a result, central nervous system (CNS) dysfunctions might ultimately be induced.

Long et al. [19–20] first revealed in their in vitro studies that TiO_2_ NPs can induce dose- and time-dependent elevations in H_2_O_2_ levels in BV2 cells (an immortalized brain microglia cell line). BV2 internalized TiO_2_ NPs and subsequently swollen mitochondria were detected by transmission electron microscopy (TEM), indicating that the function of the mitochondria was disrupted. Mitochondria are the sites of aerobic respiration, and generally are the major energy production center in eukaryotes. Dysfunction of mitochondria would thus influence energy metabolism.

Many in vivo studies have also verified the role of OS in TiO_2_ NPs-induced neurotoxicity. Wang et al. [21] found that nasal instillation of TiO_2_ NPs can lead to histopathological changes in the mouse brain. At the same time, the activity of superoxide dismutase (SOD) was inhibited, methane dicarboxylic aldehyde (MDA) levels were increased, and acetylcholin esterase (AChE) activity was enhanced in brain tissues. These changes indicated that OS was involved in neurological lesions induced by TiO_2_ NPs. In further studies, Krawczynska et al. [22] injected rats with TiO_2_ NPs intravenously. Twenty-eight days after injection, the level of aromatase was reduced and glutathione peroxidase and reductase activities were suppressed, implying that the regulation of oxidative stress in the brain was disturbed. In this study, the angiotensin system was disrupted as well. In addition, after Shrivastava et al. [23] treated male mice with TiO_2_ NPs through oral administration for 21 days, ROS increased, and the activities of anti-oxidant enzymes (such as SOD, and CAT, among others) were affected in the brain tissues. These changes were associated with neurotoxicity of NPs. TiO_2_ NPs were also shown to induce elevated levels of H_2_O_2_ and MDA in the brain after mice were put in chambers with a steady flow of TiO_2_ NPs (mimicking inhalation exposure), for 8 h per day, for 3 weeks [24]. Ma et al. [25] found that exposure to TiO_2_ NPs, through delivery in the abdominal cavity, can lead to histopathological changes in mouse brain. This was accompanied by elevated levels of ROS, MDA, constitutive nitric oxide synthase (cNOS), induced nitric oxide synthase (iNOS), and nitric oxide (NO), and inhibited activities of SOD, CAT, ascorbate peroxidase (APX), glutathione peroxidase (GSH-Px), total antioxidant capacity (T-AOC), and AChE, as well as through reduced ratios of ascorbic acid (AsA) to oxidized AsA (DAsA) and glutathione (GSH) to oxidized glutathione (GSSG). These changes implied that OS, induced by TiO_2_ NPs, mainly contributed to neurotoxicity. In addition, maternal exposure to TiO_2_ NPs was also shown to affect the OS status in the fetal brain. After pregnant rats were administrated with TiO_2_ NPs through subcutaneous injection, neonates showed down-regulated expression of CAT, GSH-Px, T-AOC, and an increase in both MDA expression and oxidative impairment of the DNA. When neonates matured, their performance was poor in behavioral tests (novel object recognition test, forced swim test, and sucrose preference test) [26].

#### Inflammatory response

Inflammatory response induced by TiO_2_ NPs is another major mechanism of neurotoxicity. When TiO_2_ NPs are transported to the brain, they interact with neurons and glial cells. Microglia are considered to be innate immune cells residing in brain. Once they are activated by exogenous substances, pro-inflammatory cytokines are released to induce neuro-inflammation [27–28]. TiO_2_ NPs acting as a stimulus were able to activate microglia cells. Su et al. [29] treated mice with TiO_2_ NPs by nasal administration for nine months, after which the glial cells showed over-proliferation and tissue necrosis was found in hippocampal area. Meanwhile, the expression of genes associated with neurotrophin signaling pathways, such as nerve growth factor (NGF) and brain-derived neurotrophic factor (BDNF), were altered in the hippocampal area, indicating that these genes were related to the neuro-inflammatory responses induced by TiO_2_ NPs. After mice were exposed to TiO_2_ NPs through intranasal administration for 90 days, expression of toll-like receptor (TLR2), TLR4, nuclear factor-kappa B (NF-κB), and tumor necrosis factor-α (TNF-α) in the mouse hippocampus was promoted. At the same time, histopathological changes were observed in the hippocampus; over-proliferation of glial cells, impaired nuclei, and cellular degeneration were observed, all of which contributed to neuro-inflammation. In addition, locomotor activity in these mice was affected. These changes suggested that inflammatory responses play a role in TiO_2_ NPs-induced neurotoxicity [30]. Ze et al. [31] treated mice with TiO_2_ NPs by intranasal administration for 90 days to determine if the p38-nuclear factor-E2-related factor-2 (p38-Nrf-2) signaling pathway was implicated in OS. Data showed that the expression of p38, Jun N-terminal kinase (JNK), NF-κB, Nrf-2, and heme oxygenase (HO-1) was promoted in the brain of TiO_2_ NPs-treated groups. Simultaneously, levels of O^2−^, H_2_O_2_, MDA, carbonyl, and 8-hydroxy-2'-deoxyguanosine (8-OHdG) were also enhanced. These findings indicated that the activated p38-Nrf-2 signaling pathway could induce excessive OS, leading to over-proliferation of spongiocytes and hematencephalon. Exposure to TiO_2_ NPs through tracheal administration has also been shown to induce the expression of interleukin-1β (IL-1β), TNF-α, and IL-10 in the brain. Herein, an impairment of the the blood–brain barrier and damage of astrocytes was observed [32].

#### Apoptosis dysfunction

Apoptosis, also called programmed cell death, is defined as the genetically determined elimination of cells. The activation of caspase plays a pivotal role in apoptosis. Human health and disease can be modulated by apoptosis [33–34]. Sheng et al. [35] found that TiO_2_ NPs induced apoptosis in primary hippocampal neurons. Elevated levels of Ca^2+^, cytochrome c, Bax, caspase-3, and caspase-12, as well as a reduction in mitochondrial membrane potential (MMP) and blc-2 levels, indicated that mitochondria- and endoplasmic reticulum-mediated signaling pathways were involved in the apoptotic process. TiO_2_ NPs were also shown to decrease cell viability by inducing apoptosis in the microglia N9 [36] and human astrocytes-like astrocytoma U87 cell lines [37].

#### Direct toxic effects on cell structures

Cell components, such as the cell membrane and mitochondria, can be targets of TiO_2_ NPs. TiO_2_ NPs can decrease cell viability of primary rat astrocytes. Herein, the mitochondrial morphology was changed and mitochondrial membrane potential (MMP) was reduced, suggesting mitochondrial impairment. At the same time, glutamate uptake was down-regulated, and ROS was promoted [38]. Coccini et al. [39] found that when D384 (human glial cell line) and SH-SY5Y (human neuronal cell line) cells were treated with TiO_2_ NPs, mitochondrial dysfunction, impaired cell membrane, and changes in cell morphology were detected. Mao et al. [40] discovered that a dose of TiO_2_ NPs, having no effect on viability in SH-SY5Y cells could cause changes in cell morphology and disruptions to the microtubule structure, both of which are associated with neurotoxicity. In addition, Ben Younes et al. [41] treated rats with TiO_2_ NPs through intraperitoneal injection. After which, rats exhibited altered emotional behavior in a plus maze test; however, histopathological examination demonstrated no significant differences between treated and control groups. The study failed to discuss other mechanisms associated with nanoneurotoxicity.

#### Genotoxicity

Genotoxicity is simply defined as the induction of DNA damage, in a direct or indirect manner, caused by substances such as benzopyrene in cigarettes or some chemotherapeutic drugs. In vivo and in vitro studies typically measure genotoxicity using the comet assay, the micronucleus test, the Ames test, and the chromosome aberration assay [42]. Golbamaki et al. [42] summarized genotoxicity data of NPs from available studies in their review, concluding that NPs can induce genotoxicity, and the mechanisms through which this occurs can be divided into direct primary genotoxicity, indirect primary genotoxicity, and secondary genotoxicity (for a comprehensive review, see Golbamaki et al. [42]). TiO_2_ NPs, like other types of engineered NPs, can induce genotoxicity. However, Golbamaki et al. did not report on TiO_2_ NPs-induced genotoxicity in the brain or in brain cells. Obviously, the relationship between neurotoxicity of TiO_2_ NPs and genotoxicity should be investigated comprehensively. Recently, El-Ghor et al. [43] determined that TiO_2_ NPs could cause DNA damage in the mouse brain. This genotoxicity could be alleviated by co-treatment with chlorophyllin (CHL). CHL is a free radical scavenger and is able to reduce the harmful effects of OS [44–45]. These findings suggest that oxidative stress induced by TiO_2_ NPs can cause genotoxicity to the brain.

#### Multiple mechanisms

Usually, activation of glia cells and mitochondrial injury are able to initiate excessive ROS production [27,46]. Meanwhile, ROS can lead to apoptosis and genotoxicity [43,47]. Therefore, the above-mentioned mechanisms, including OS, apoptosis, inflammatory responses, genotoxicity, and direct impairments on cell components may be jointly implicated in TiO_2_ NPs-induced neurotoxicity. OS (which promotes ROS and affects the activities of SOD, GPx, CAT) and mitochondrial impairments were observed in TiO_2_ NPs-treated glial cells (C6 and U373) [48]. After glial cells are damaged by TiO_2_ NPs, inflammatory responses would presumably occur. This might exacerbate brain damage further. Wang et al. [49] found that intranasal instillation of TiO_2_ NPs can induce histopathological changes in the mouse brain, in which OS (MDA increases) and inflammatory responses (elevated expressions of TNF-α and IL-1β) were involved. Shimizu et al. [50] analyzed the brains of mouse offspring by cDNA microarray and found that prenatal exposure to TiO_2_ NPs could alter the expression of neurotransmitter genes as well as genes associated with apoptosis, OS, and psychiatric disorders. TiO_2_ NPs decreased cell viability in PC12 cells in a dose- and time-dependent manner by increasing the level of ROS and proportion of apoptotic cells. Pretreating PC12 with a ROS scavenger could alleviate these harmful effects induced by TiO_2_ NPs [51]. In addition, an inhibition in cell proliferation, altered cell morphology (assessed by decreased F-actin), and apoptosis could be induced by TiO_2_ NPs in C6 and U373 cells. TiO_2_ NPs were also internalized by C6 and U373 cells [52]. Valdiglesias et al. [53] found that NPs were internalized by SH-SY5Y neuronal cells exposed to TiO_2_ NPs, which coincided with alterations in the cell cycle and an elevation in the proportion of apoptotic cells. Damage of the DNA was induced and NO oxidative stress was observed in these experimental groups. The treatment of mice with TiO_2_ NPs by intragastric administration resulted in an impairment of their spatial recognition memory. This impairment was mainly due to elevated expression of caspase-3 and caspase-9, Bax, and cytochrome c, and suppressed Bcl-2, in the hippocampal area. Meanwhile, the ROS levels were enhanced, and the activities of antioxidant enzymes such as SOD, CAT, ascorbate peroxidase (Apx), and GSH-Px were inhibited. In addition, the ratios of AsA to DAsA and GSH to GSSG were decreased. These changes suggested that apoptosis and OS were involved in TiO_2_ NPs-induced neurotoxicity [54]. Meena et al. [55] found that after rats were administrated TiO_2_ NPs by intravenous injection, once a week for four weeks, the ROS in the brain were significantly enhanced. This elevation of ROS promoted an inflammatory responses and led to decreased activities of SOD and GPx, elevated MDA and DNA damage, as well as an increased proportion of apoptotic cells.

#### Minor mechanisms

In addition to major mechanisms of TiO_2_ NPs-induced neurotoxicity, other minor mechanisms exist. Disdier et al. [6] discovered that after rats received TiO2 NPs through intravenous injection, neuro-inflammation was not directly induced by Ti accumulation in the brain, but instead was indirectly stimulated by cytokines or pro-inflammatory mediators in systemic circulation. Hong et al. [56] demonstrated that the decreased cell viability of primary hippocampal neurons was associated with inhibited dendritic growth, disrupted glutamate metabolism, dysregulated levels of *N*-methyl-D-aspartate receptors (NMDA receptors), increased Ca^2+^ levels and voltage of I_k_ in cells, and reduced activity of ATPase. However, additional experiments were needed to further discuss how each of these parameters contributed to neurotoxicity. Ze et al. [57] found that after mice were treated with TiO_2_ NPs by oral exposure, histopathological changes in the hippocampus were occurred. Meanwhile, affected neuron ultrastructures, such as swollen mitochondria and impaired nuclear membrane were detected. Long-term potentiation (LTP) in the hippocampus was reduced and the expressions of NMDA receptors were down-regulated as well. These changes contributed to impaired spatial memory. Prenatal exposure to TiO_2_ NPs was also shown to result in decreased cell proliferation in the hippocampus of rat offspring, which was associated with poor performance in the Morris water maze test and passive avoidance test [58]. Maternal exposure to TiO_2_ NPs can also affect the production of dopamine and its metabolites, as determined by high performance liquid chromatography (HPLC) [59], and alter gene expression related to dopamine systems, as measured by DNA microarray in neonatal mouse brain [60]. Mice performed poorly in the Y-maze test after a 60 d exposure to TiO_2_ NPs, and histopathological changes were observed in brain. Meanwhile, the intracellular content of trace elements (Ca, Mg, Na, K, Zn, and Fe) was disturbed. The activities of Na^+^/K^+^-ATPase, Ca^2+^-ATPase, and Ca^2+^/Mg^2+^-ATPase also decreased, and the levels of neurotransmitters, including acetyl choline (Ach), glutamic acid (Glu), and NO were elevated. The expression of monoamine neurotransmitters consisting of norepinephrine (NE), dopamine (DA), dihydroxyphenylacetic acid (DOPAC), 5-hydroxytryptamine (5-HT), and 5-hydroxyindoleacetic acid (5-HIAA) was down-regulated. These changes impaired spatial recognition memory [61]. When mother rats were exposed to TiO_2_ NPs during the lactation period, their offspring exhibited dysregulated synaptic plasticity that included the input/output (I/O) function, paired-pulse reaction, and long-term potentiation in the hippocampal zone. These developmental changes in the hippocampus could impair learning ability and memory [62]. TiO_2_ NPs also decreased PC12 viability through activation of JNK- and p53-mediated pathways, which disrupted cell cycle, leading to apoptosis and excessive ROS [63].

### Uncertain mechanisms underlying TiO_2_ NPs-induced neurotoxicity

#### Autophagy

Autophagy can be divided into three types, 1) microautophagy, 2) macroautophagy, and 3) identified-chaperone-mediated autophagy. It is believed that macroautophagy (referred to as autophagy) is the main degradative pathway among these subtypes of autophagy [64–65]. Autophagy also acts as a dynamic recycling system in which new building blocks, and energy for cellular renovation and homeostasis, are produced [12]. However, excessive autophagy can contribute to cell death. Autophagy has been determined to be a potential mechanism of nanotoxicity [9,66]. However, few studies have described the relationship between neurotoxicity and nanomaterials. It was revealed that gold nanoparticles can increase the levels of autophagy-related proteins in human lung fibroblasts (MRC-5), concomitant with excessive MDA production [67]. After lung epithelial cancer cells (A549) were exposed to iron oxide nanoparticles, ROS production, mitochondrial impairments and autophagy were detected [68]. Autophagy in human peripheral blood monocytes can be induced by cerium dioxide nanoparticles [69]. In addition, copper oxide NPs [70], silica NPs [71], zinc oxide NPs [72], and silver NPs [73] were shown to induce autophagy in in vitro studies. TiO_2_ NPs were also capable of inducing autophagy. Studies showed that TiO_2_ NPs could induce autophagy in normal lung cells [74] and in primary human keratinocytes [75]. Based on the studies illustrated above, we hypothesize that TiO_2_ NPs can induce autophagy dysfunctions in brain tissues and cells. Therefore, autophagy could be a potential mechanism underlying TiO_2_ NPs-induced neurotoxicity. However, more studies are needed to further investigate the relationship between brain damage and TiO_2_ NPs-mediated autophagy dysfunction.

#### Epigenetics

Another potential mechanism of TiO_2_ NPs is epigenetic regulations. Epigenetic changes were reported to be implicated in nanotoxicity [10–11]. Epigenetics refers to all heritable alternations in gene expression not caused through changes to the DNA sequence itself, but rather arise during development and cell proliferation [13–14]. In other words, the epigenetics represents the interactions between gene expression and environmental factors, and the relationship between genes, which were modulated during development and adult life [76]. In most situations, epigenetic modifications modulate DNA transcription through mechanisms such as DNA methylation [77], histone modiﬁcations [78], and non-coding RNA (ncRNA) regulation [79].

Among them, DNA methylation is the most extensively studied epigenetic mechanism, wherein a methyl group (-CH_3_) from *S*-adenosylmethionine (SAM) is transferred to the 5-position of cytosines, in certain CpG dinucleotides, by a family of DNA methyltransferase enzymes (DNMTs). The DNMTs can be classified into three major types based on their different structures and functions, DNMT1, DNMT3a, and DNMT3b [77,80–82]. DNMT1 was identified as having a role in the maintenance of methylation during each cellular replication when DNA is duplicated [83]. DNMT3a and DNMT3b have de novo methylation ability, wherein new 5-methylcytosines are introduced in initially non-methylated genome sites [84–85]. In most cases, DNA methylation not only induces gene silencing [86], but also is related to the initiation of DNA replication [87], DNA mismatch repair [88] and inactivation of transposons [89].

Recently, more and more studies have discovered that CNS dysfunction may be potentially affected by DNA methylation. DNA methylation was associated with expression of neurotransmitters. In an in vitro study, hypermethylation of the excitatory amino acid transporter (EAAT2) promoter in glioma cells led to a deficiency in astroglial EAAT2 expression, which was related to the pathogenesis of CNS disorders with remarkable excitatory toxicity elements. Furthermore, the transcription of EAAT2 could be recovered by suppression of DNA methyltransferases [90]. Neurodegenerative diseases, such as Alzheimer's disease (AD) [91], Huntington’s disease (HD) [92], and amyotrophic lateral sclerosis (ALS) [93], are regulated by DNA methylation. Meanwhile, psychiatric disorders have been associated with an abnormal DNA methylation status in brain [94–96].

Few reports have described abnormal DNA methylation induced by NPs. SiO_2_ NPs can reduce global DNA methylation levels and change the methylation status of the PARP-1 promoter in human keratinocytes (HaCat) [97–98]. Silver NPs had the ability to change DNA methylation as well [99]. In a recent study, DNA methylation status, regulated by DNMTs in the A549 cell line, could be altered by oxidative stress induced by TiO_2_ NPs [100]. As OS is a main mechanism of nanotoxicity, we hypothesize that TiO_2_ NPs might be able to alter DNA methylation status in the brain through an OS-mediated pathway. More studies are needed to further investigate the role of DNA methylation in TiO_2_ NPs-induced neurotoxicity.

## Conclusion

TiO_2_ NPs possess unique characteristics due to their tiny size and are widely used in many fields. The rapid development of nanotechnology and widespread applications of products based on nanomaterials are possible causes of neurological disorders in humans. Therefore, numerous in vivo and in vitro studies have been conducted to assess the neurotoxicity of TiO_2_ NPs. However, the exact underlying mechanisms of TiO_2_ NPs-induced neurotoxicity are unclear. Through summarizing studies describing the neurotoxicity of TiO_2_ NPs, we found that these mechanisms mainly consisted of oxidative stress (OS), apoptosis, inflammatory responses, genotoxicity, and direct impairment of cell components. Meanwhile, other mechanisms, including disturbed distributions of trace elements, disrupted signaling pathways, dysregulated levels of neurotransmitters, and synaptic plasticity also contribute to the neurotoxicity of TiO_2_ NPs. Furthermore, recent studies implicated autophagy and DNA methylation as results of nanotoxicity. Therefore, we hypothesized that these two mechanisms could be potentially involved in the neurotoxicity of TiO_2_ NPs. Further studies are needed to test these hypotheses.

In summary, to fully understand the health threats to the brain posed by TiO_2_ NPs, and improve the bio-safety of TiO_2_ NPs-based products, every possible molecular mechanism of TiO_2_ NPs-induced neurotoxicity must be investigated comprehensively.
